# Robust Position-Only Null Steering in Linear Antenna Arrays via a Nature-Inspired Optimizer for Wireless Communication

**DOI:** 10.3390/biomimetics11050304

**Published:** 2026-04-27

**Authors:** Ali Yildiz, Ali Akdagli, Filiz Karaomerlioglu, Gökhan Yüksek, Davut Izci, Vedat Tümen, Serdar Ekinci, Mohammad Salman, Mohammad Al-Rabayah

**Affiliations:** 1 Department of Electrical and Electronics Engineering, Mersin University, 33150 Mersin, Turkey; yildiz@mersin.edu.tr (A.Y.); akdagli@mersin.edu.tr (A.A.); filizkrm@mersin.edu.tr (F.K.); 2 Department of Electrical and Electronics Engineering, Batman University, 72100 Batman, Turkey; gokhan.yuksek@batman.edu.tr; 3 Department of Electrical and Electronic Engineering, Bursa Uludag University, 16059 Bursa, Turkey; davutizci@uludag.edu.tr; 4 Department of Computer Engineering, Bitlis Eren University, 13100 Bitlis, Turkey; sekinci@beu.edu.tr; 5 College of Engineering and Technology, American University of the Middle East, Egaila 54200, Kuwait; mohammad.alrabayah@aum.edu.kw

**Keywords:** position-only synthesis, null steering, linear antenna arrays, honey formation optimization, HFOSC, adaptive beamforming, interference mitigation, metaheuristic optimization, wireless communications, array pattern synthesis

## Abstract

The demand for hardware-efficient interference suppression algorithms is growing with the increasing density in wireless communication networks. In this paper, a robust position-only null steering method for linear antenna arrays is proposed based on Honey Formation Optimization with Single Component (HFOSC), a metaheuristic algorithm founded on the ripening process of honey in beehives. By optimizing only the element locations, the proposed method avoids the use of phase shifters and attenuators, thus reducing implementation complexity while maintaining flexibility in pattern control. A 30-element linear array with Chebyshev excitation is used to test the technique under representative interference scenarios such as single-null, multiple-null, and wide-sector nulling cases, as well as constrained practical designs. The simulation results demonstrate that the proposed approach can realize strong interference suppression across different cases while maintaining the main-beam shape and acceptable sidelobe performance. In idealized discrete-interference cases, nulls below −90 dB are achieved, while in a practical constrained design with a minimum inter-element spacing of 0.5λ and a position resolution of 0.1λ, a null depth of −72.89 dB is still achieved, confirming the practical applicability of the method. Moreover, comparative results with GA, PSO, and DE over 100 independent runs illustrate that HFOSC achieves the lowest optimization cost and the smallest standard deviation, along with a favorable overall trade-off between beam preservation and null suppression, with statistically significant superiority in optimization performance. The proposed method does not require phase shifters and attenuators, providing a simple, hardware-friendly, and robust solution for adaptive interference cancellation in wireless communication systems.

## 1. Introduction

With the fast development of 5G and the emergence of next-generation 6G wireless networks, system-level requirements such as spectral efficiency, ubiquitous connectivity, higher data rates, and ultra-low latency have been significantly tightened. This places unprecedented demands on the radio frequency front-end, which must employ sophisticated spatial interference suppression techniques [1,2,3,4]. In this era of spectral congestion, the ability to spatially cancel undesired signals is as important as concentrating energy on the desired user. Consequently, adaptive beamforming and null steering are the pillars of contemporary wireless communication systems [5,6,7,8].

Recent progress in wireless communication has investigated different architectures to address the increasing requirements of next-generation networks. The integration of satellite–terrestrial networks (STNs) has been widely examined to enhance coverage and reliability, for example, in the context of 6G ubiquitous connectivity. Zhou et al. [9] proposed a hierarchical deep learning-based mission-driven resource scheduling scheme for STNs by leveraging resource collaboration and reconfiguration to maximize mission accomplishment under dynamic resource disturbances. Coupled with this, the Constellation as a Service model presented in [10] considers the entire multi-constellation architecture as a shared resource pool from which optimal sub-constellations can be formed dynamically for each direct-satellite-to-device service region using predictive satellite beamforming and pre-configured handover paths, resulting in improved satellite service rates and reduced handover overhead. Furthermore, Gao et al. [11] proposed a LEO satellite-assisted deep space communication system employing the decode-and-forward relaying protocol, wherein the FSO link is characterized by the exponentiated Weibull distribution to account for pointing error impairments, coronal turbulence, and solar noise, while the terrestrial link is modeled using the shadowed-Rician distribution. The joint pseudo-range and Doppler positioning method proposed in [12], leveraging signals of opportunity from LEO constellations such as Starlink and Iridium NEXT, further underscores the versatility of satellite-based systems as a viable complement to conventional GNSS services.

In parallel, innovative antenna array designs have been developed to enhance bandwidth and scanning capabilities. A miniaturized low-profile ultrawideband antipodal Vivaldi antenna (AVA) array loaded with edge techniques was presented in [13], achieving a measured operating band of 0.9–12 GHz with active VSWR less than 3 when scanning up to ±60° in both E- and H-planes. Moreover, an ultralow-profile dual circularly polarized beam-reconfigurable array antenna based on the Risley prism beam-scanning principle was proposed in [14], combining a large aperture feed array with a phase-gradient transmissive metasurface, making it well suited for satellite communication applications. The use of intelligent reflecting surfaces (IRS) has also shown considerable promise in uncrewed aerial vehicle (UAV) covert communications [15], where joint optimization of UAV trajectory, transmit beamforming, and IRS phase shift matrix via block coordinate descent substantially extends covert transmission range and robustness. Advanced beamforming techniques for non-circular signals have further been studied to cope with complex interference scenarios, with Meng et al. [16] proposing a widely linear beamforming framework in coprime arrays with non-uniform noise, enabling accurate direction estimation and interference-plus-noise covariance matrix reconstruction through atomic norm minimization with Toeplitz and Hankel constraints. In parallel, the design of energy-selective antennas has gained attention for simultaneous electromagnetic protection and interference suppression [17].

Even though a very rich set of solution methods is presented, most of the material discussed, from satellite–terrestrial integration, relay-assisted deep space links, wideband array design, covert UAV communications, to advanced beamforming of non-circular signals, converges into a common and increasingly important problem in closely spaced network setups: maintaining strong directional beams toward desired users while simultaneously canceling interference from unwanted ones through precise null placement. With the ever-increasing network densities and an increasingly crowded electromagnetic environment, steerable beams and time-varying nulling become common features for all the above-mentioned system architectures, and a new class of non-stationary problems arises, which attracts interest toward developing unified and robust beamforming solutions capable of practical realization under array perturbations, non-stationary noise, and non-circular signal statistics.

Solving this problem requires antenna designs that are structurally simple yet highly adaptable. In this respect, linear antenna arrays are widely used as suitable candidates because they can be simply realized, the beam can be accurately steered, and they are regarded as building blocks of complex array architectures in recent years. However, effective utilization requires that the beams formed by the transmitter and receiver be aligned, and this in turn requires that the antenna arrays be able to adaptively form deep nulls to protect quality of service, reduce co-channel interference, and provide an acceptable signal-to-interference-plus-noise ratio. Such a constraint becomes critical in dense wireless communication networks, since spectral efficiency and spatial reuse are highly desirable. Moreover, if the directions of arrival of interferers are unknown and/or time-varying, the demand for radiation patterns featuring deep and robust nulls becomes even more compelling [4,8,18,19,20,21].

To address this need, various null steering techniques have been developed over the years, which can be classified according to which array parameter is varied. Even though complex weight control offers a better degree of flexibility, it is necessary to employ variable attenuators and phase shifters on each element, which increases the complexity of implementation and calibration. A simpler method is the amplitude-only control method, but it introduces significant trade-offs, as it inherently reduces the overall array gain and power efficiency due to the dissipation of power in attenuators, and often distorts the main lobe shape by broadening the beamwidth and increasing sidelobe levels [22]. Phase-only control is more power efficient than amplitude-only techniques; however, it also has its own problems, such as complicated nonlinear optimization, main lobe and sidelobe distortions, hardware-related phase quantization errors, and constrained degree of freedom in realizing arbitrary deep null relocation without incurring pattern distortion [6,7,21].

Position-only control, which consists of manipulating the position of the elements of the array, is becoming an option with interesting advantages. Due to symmetry, symmetrically placed elements produce natural symmetric nulls which greatly eases the steering process and improves system performance. The approach exhibits no dependency on phase shifters and therefore can lead to further research wherein the phase shifters could be exclusively dedicated to main beam steering, and the old approach of user tracking-based interference rejection can continue from a place separate from user tracking. Moreover, the amplitude distribution can be separately optimized for certain sidelobe distributions, which adds more freedom to the beam pattern design. From the standpoint of hardware, the technique is very hardware-friendly as it eliminates the need for lossy RF components such as variable attenuators, and, in particular, is able to address high-power applications. Since at millimeter-wave frequencies the small wavelengths allow for mechanical positioning systems with a sufficiently small step size and reasonably sized actuators, this provides a low-cost and power-efficient solution scalable for high-frequency wireless communication applications [23,24,25,26].

Synthesizing element locations for null steering is a multidimensional, multi-modal parameter search problem. Traditional gradient-based approaches have proved to be inadequate for such problems, as they are prone to becoming trapped in local minima and often fail to yield satisfactory solutions. As a result, AI-based metaheuristic optimization techniques such as Genetic Algorithms (GA) [27], Particle Swarm Optimization (PSO) [28], and Differential Evolution (DE) [29] have gained considerable popularity in antenna array synthesis and null steering applications [24,25,30,31,32,33,34,35,36,37].

Another recently developed member of the family of nature-inspired heuristics is honey formation optimization (HFO), which simulates the gradual maturation of honey in a beehive [38,39]. The classical HFO was developed as a multi-component model. A further simplification led to a single-component version, named HFO-1, in which all candidate solutions evolve independently [40]. For convenience, this variant is referred to herein as honey formation optimization with single component (HFOSC). The HFOSC method was thoroughly tested against 60 benchmark test functions. The findings illustrate that HFOSC achieves better results than well-known metaheuristics, including PSO, Differential Evolution, Moth-Flame Optimization, Whale Optimization Algorithm (WOA), and the Improved Grey Wolf Optimizer, in terms of solution accuracy and convergence rate [40].

The main contributions of this paper consist of an HFOSC-based implementation and evaluation of position-only null steering for a 30-element Chebyshev linear array under several representative scenarios. This includes the formulation of a multi-objective cost function that simultaneously accounts for null depth, sidelobe level, and pattern fidelity; validation across single-null, multi-null, and wide-sector cases; a positional quantization study; and an additional constrained design example that reflects practical hardware limitations. The advance is scoped at the optimizer level within the established position-only null-steering framework: the paper demonstrates the applicability and comparative advantage of HFOSC for this class of problems rather than proposing a new null-steering paradigm.

The rest of the paper is organized as follows: Preliminaries of the array model and the cost function are explained in Section 2. Section 3 describes the HFOSC-based optimization and the methodology. Section 4 illustrates design examples and numerical results to verify the effectiveness of the proposed approach across representative cases, such as discrete nulling, positional quantization analysis, wideband sector nulling, and practical issues related to mutual coupling effects and position resolution constraints. Section 5 reports a comparative performance evaluation of HFOSC with other well-known metaheuristic algorithms. Finally, conclusions and future work are presented in Section 6.

## 2. Mathematical Formulation

### 2.1. Array Factor and Position Perturbation

Consider a linear antenna array with (2N) isotropic radiators spaced symmetrically about the center of the array. The far-field array factor in the azimuth plane, for an observation angle *θ* ∈ [−90°, 90°] measured with respect to the broadside, is given by:

(1) $$AF(\theta )=\sum_{n=-N}^{N}a_{n}e^{jkd_{n}\sin\theta }\;\;\;\;\;\;\;\;\;-90{}^{\circ}\leq \theta \leq 90{}^{\circ}$$

In the array factor expression, parameters include the excitation amplitude $a_{n}$ of the n-th element, the position *d_n_* of the element measured from the array center, the wavenumber k = 2π/λ (where λ is the wavelength), and the observation angle *θ*. For symmetric arrays with real excitation coefficients ($a_{-n}=a_{n}$) and symmetric positioning ($d_{-n}={-d}_{n}$) with *n* = ±1, ±2, …, ±*N*, the array factor simplifies to:

(2) $$AF\left(\theta \right)=2\sum_{n=1}^{N}a_{n}\cos\left(kd_{n}\sin\theta \right)$$

In the above formulation, each array element is modeled as an isotropic radiator, i.e., EF(θ)=1 for all θ, where EF(θ) is the element factor representing the radiation pattern of an individual array element. Although isotropic radiators are physically unrealizable, this assumption is standard practice in array signal processing literature to isolate the array’s spatial filtering behavior from element-specific radiation characteristics, noting that practical elements such as microstrip patch or dipole antennas introduce amplitude tapering that can alter null depths and positions, particularly at wide scan angles. Under this assumption, and with broadside operation under uniform zero-phase excitation (β=0), the array factor is real-valued. In the position-only nulling method, the excitation amplitudes are held constant and are chosen based on a target initial pattern, with a Chebyshev distribution for equal sidelobes being a typical example, while the element locations serve as the sole variables to be optimized. The contribution of each element to null generation is then controlled by applying position perturbations $\delta_{n}$ such as:

(3) $$d_{n\_new}=d_{n\_initial}+\delta_{n}$$

where $d_{n\_initial}$ represents the initial location of Chebyshev array elements and *δ_n_* is the position perturbation to be optimized by the HFOSC algorithm.

### 2.2. Optimization Cost Function

The generation of the desired array radiation pattern with prescribed nulls is posed as a multi-objective constrained optimization problem, and an efficient cost function must consider competing objectives such as pattern fidelity, which involves remaining close to the desired initial pattern (e.g., Chebyshev), null depth level (*NDL*), which requires providing sufficient suppression at interfering directions, and maximum sidelobe level (*MSL*), which aims to limit the sidelobe level outside null regions. The overall cost function is given as:

(4) $$C=\sum_{\theta =-90^{{}^{\circ}}}^{90^{{}^{\circ}}}\left|AF_{o}\left(\theta \right)-AF_{d}\left(\theta \right)\right|+ENDL+EMSL$$

where *AF*_o_(*θ*) is the array factor of the current solution, *AF*_d_(*θ*) is the desired array factor, *ENDL* and *EMSL* are the enforcement terms for the null depth and the sidelobe levels, respectively.

The desired pattern is defined piecewise to localize nulling requirements:

(5) $$AF_{d}\left(\theta \right)=\left\{\begin{array}{l} 0,\;\;\;\;\;\;\;\;\;\;\;\;\;\;\;\;\;\;\;\;\;\;\;\;\;\;\;\;\;\;\;\;\;\;\;\;\;\;\;\;\;\;\mathrm{if}\;\theta =\theta_{na}\\ \mathrm{Initial}\;\mathrm{Chebyshev}\;\mathrm{pattern},\;\mathrm{otherwise} \end{array}\right.$$

where *θ_na_* represents the null angle. Such a definition is very selective in the sense that it guarantees that only the desired directions are nulled, while the rest of the pattern still maintains a very high similarity to the original Chebyshev pattern. To avoid unnecessary penalization once sufficient null depth is achieved, a threshold-based error term is introduced:

(6) $$ENDL=\left\{\begin{array}{l} 0,\;\;\;\;\mathrm{if}\;AF_{0}\left(\theta_{na}\right)\leq NDL\\ 1,\;\;\;\;\mathrm{otherwise} \end{array}\right.$$

This prevents the cost function from penalizing solutions that already achieve the desired suppression level. To prevent sidelobe level increases during null steering, the following enforcement function is applied only for angular positions outside the main lobe:

(7) $$EMSL=\left\{\begin{array}{ll} 0, & \mathrm{if}\;|AF_{o}(\theta )|\leq MSL,|\theta |>\theta_{ML}/2\\ 1,\;\; & \mathrm{otherwise} \end{array}\right.$$

where $\theta_{ML}$ is the main lobe width, and *MSL* and *NDL* are the thresholds specified in dB. In the numerical implementation, $|AF_{o}(\theta )|$ is evaluated in dB scale to ensure dimensional consistency with these thresholds. The binary enforcement terms $E_{NDL}$ and $E_{MSL}\in \{0,1\}$ are intentionally chosen as hard constraints to prevent any null depth or sidelobe level violation beyond the prescribed thresholds, at the cost of a non-smooth penalty landscape. Since the determination of the element positions constitutes a nonlinear constrained optimization problem with multiple local minima, metaheuristic solution approaches are particularly well suited for this task. The following section details the HFOSC algorithm adopted in this work.

## 3. Honey Formation Optimization with Single Component (HFOSC)

**Figure 1:**
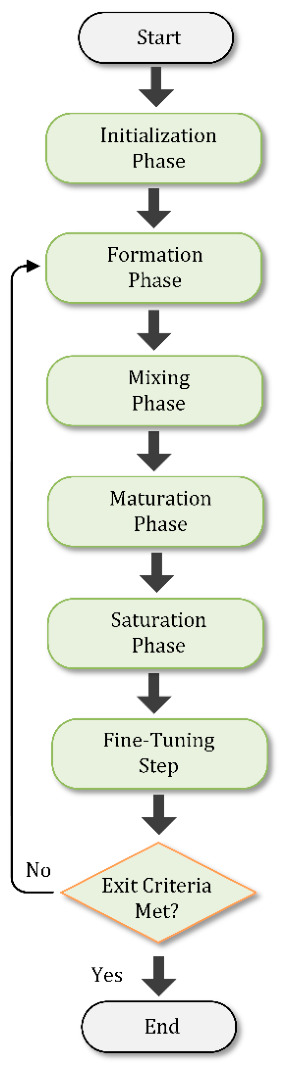
The flowchart of HFOSC algorithm.

HFOSC is a metaheuristic optimizer based on the natural phenomena of trophallaxis and enzymatic processing, similar to activities in bee colonies during honey production. The original HFO is a multi-component system; however, in HFOSC, each candidate solution is a single component, which can be compared to an entity evolving through different states of “maturation.” The algorithm is performed on a population of candidate solutions, which are element position vectors. The process is segmented into three separate mixing phases, mirroring how nectar is transformed into honey. Figure 1 shows the flowchart of the HFOSC algorithm. It proceeds through the following steps:

1. Initialization: A set of candidate solutions is randomly generated within the permissible range.
2. Iterative Refinement Loop:
  - Mixing-0 (Exploration): A local exploratory perturbation is applied to all sources in turn.
  - Mixing-1 (Refinement): Semi-mature solutions are refined by proto-mixing (a structured mixing) through exchanging information between them.
  - Mixing-2 (Exploitation): Mature solutions are exploited more intensively by recombining attributes of high-quality solutions.
3. Maturation and Saturation: Solutions that fail to progress for a number of iterations are considered mature. When the population stagnates, the best global solution (*Gbest*) is injected into the population as a stimulating agent to further enhance convergence and diversity.
4. Termination: If the maximum number of iterations is reached or the convergence criteria are satisfied, the algorithm terminates.

In HFOSC-based optimization algorithms, the selection of key parameters plays a critical role in balancing exploration and exploitation capabilities, preventing premature convergence, and controlling convergence speed. The population size (*Npop*) typically ranges from 50 to 100 individuals; while smaller populations reduce computational load, they may limit diversity, and larger populations enhance diversity but increase computational cost. For the 15-dimensional search space considered in this work, a population size in the range of 50–100 individuals provides adequate diversity while maintaining computational tractability, consistent with established practice for problems of comparable dimensionality. The maximum number of iterations (*MaxIter*) is generally set between 100 and 500, providing sufficient iterations for stable convergence without excessive runtime. The maturation period (*Nmat*), which determines how many iterations a source can remain without improvement, typically varies from 10 to 20 iterations, serving to avoid resource waste and escape local optima. The mixing probability, governing the frequency of Mixing-1 and Mixing-2 operations, generally falls within the range of 0.3 to 0.7, adjusting the trade-off between exploration and exploitation. Finally, the saturation threshold, representing the number of consecutive maturation periods without improvement in the global best solution (*Gbest*), is typically set to 2 or 3 periods, triggering diversity enhancement mechanisms only when stagnation is detected.

## 4. Design Scenarios and Numerical Results

For the validation of the position-only null steering method, a 30-element linear array (N = 15) has been synthesized, with element spacing expressed in terms of the operating wavelength λ. The original array had a 30 dB Chebyshev amplitude taper. Three different design examples corresponding to various interferer scenarios are presented. To mitigate potential mutual coupling, the minimum spacing of adjacent elements is constrained to 0.35λ.

The HFOSC parameters have been carefully selected based on preliminary experiments and computational considerations for the specific antenna array synthesis problem addressed in this study. The population size is set to *Npop* = 70 to maintain adequate population diversity while keeping the computational burden manageable. The maximum number of iterations is chosen as *MaxIter* = 200, which provides sufficient convergence opportunities given the problem’s complexity. A maturation period of *Nmat* = 15 iterations is adopted, allowing sources to mature adequately without excessive delay. The mixing probability is set to 0.5 to achieve a balanced frequency between Mixing-1 and Mixing-2 operations. The balance between exploration and exploitation in HFOSC, together with the adaptive maturing rule, is well-suited to high-dimensional, nonlinear problems such as antenna-array synthesis. Lastly, the saturation threshold is taken as 3 maturation periods, ensuring that the saturation procedure is activated only when genuine stagnation in *Gbest* is observed, thereby preventing unnecessary interventions. These parameter values are selected to ensure both the effectiveness and efficiency of the HFOSC algorithm in solving the antenna array synthesis problem. Note that all optimizations were carried out on a conventional PC (Intel Core i5, 16 GB RAM) using the HFOSC method coded in Python version 3.13.5, with angular sampling of 0.25° over the interval [0°, 90°].

### 4.1. Design Case I: Precision Suppression of Discrete Interferers

**Figure 2:**
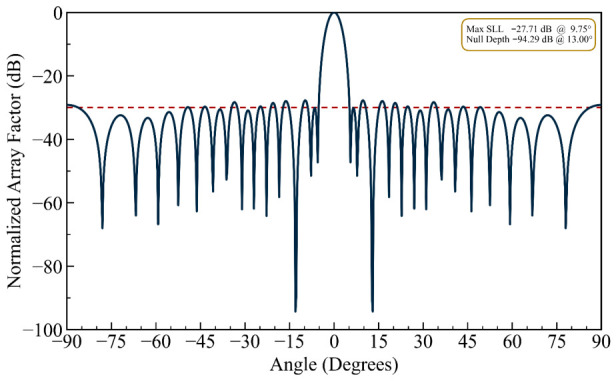
The array factor exhibiting a single null at 13.0°, corresponding to the third sidelobe (the dashed line is the sidelobe envelope).

The problem of suppressing discrete line jammers operating in the same frequency band and arriving from known directions is considered in the first design example. The first interferer eliminated was located at θ = 13.00° in the direction of the third sidelobe peak of the original pattern. The aim of the optimization was to obtain a null depth better than −90 dB. Therefore, the *NDL* and *MSL* are set to −90 dB and −27.5 dB, respectively. As shown in Figure 2, the HFOSC method successfully relocated the elements to form a sharp null of −94.3 dB at the desired direction. Due to the symmetric array configuration, an image null is also formed at −13.00°. The half-power beamwidth (HPBW) was kept fixed at 8.5°, and the *MSL* improved slightly to −27.71 dB.

**Figure 3:**
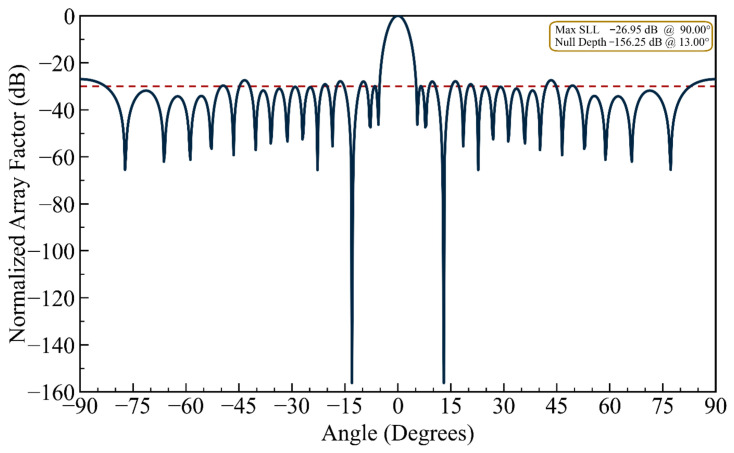
The array factor exhibiting a single null at 13.0° emphasizing enhanced null depth performance (the dashed line is the sidelobe envelope).

**Figure 4:**
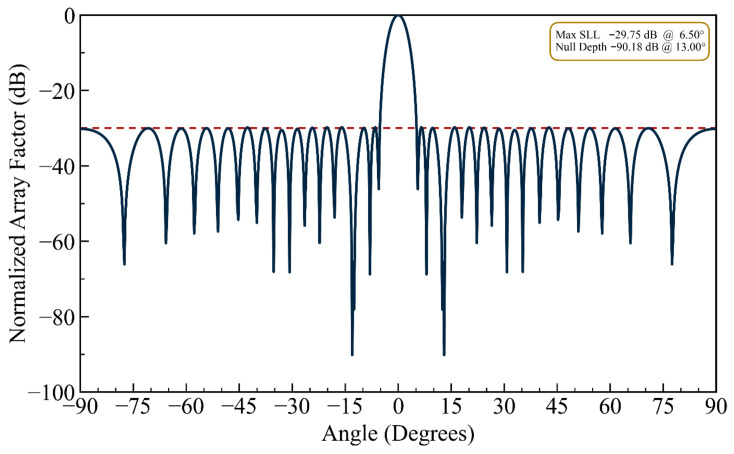
The array factor exhibiting a single null at 13.0° under a constrained maximum sidelobe level (the dashed line is the sidelobe envelope).

**Figure 5:**
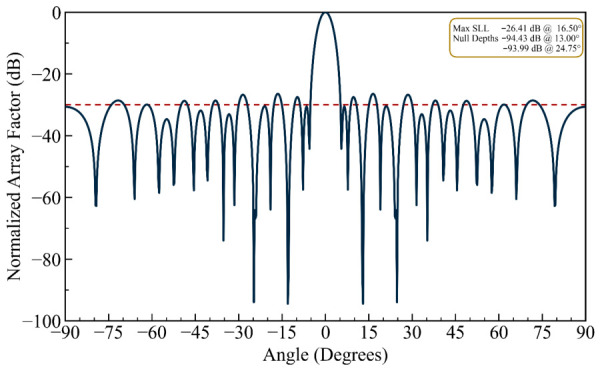
The array factor exhibiting double nulls at 13.0° and 24.75°, corresponding to the third and sixth sidelobes (the dashed line is the sidelobe envelope).

**Figure 6:**
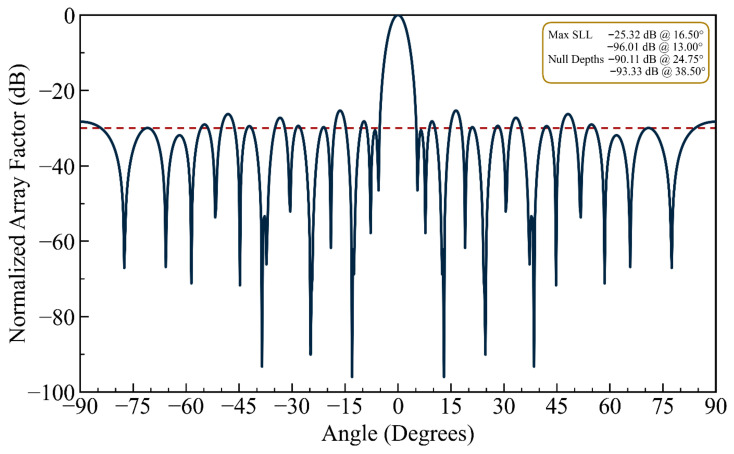
The array factor exhibiting triple nulls at 13.0°, 24.75°, and 38.5°, corresponding to the third, sixth, and ninth sidelobes (the dashed line is the sidelobe envelope).

(i) Deeper Nulling Capability: To demonstrate the optimizer’s capacity for achieving deeper nulls, the *NDL* was set to −150 dB, upon which the optimizer converged to a solution yielding a null depth of −156.3 dB, as shown in Figure 3. The *MSL* rose marginally to −26.95 dB, reflecting a modest but acceptable trade-off between null depth and sidelobe performance. While these results effectively illustrate the mathematical capability of the proposed algorithm, such extreme null depths are achievable only under idealized simulation conditions—where effects such as mutual coupling, element position tolerances, finite ground plane dimensions, and thermal noise are entirely neglected. In practice, however, the thermal noise floor alone sits at approximately −174 dBm/Hz, and even micron-level element position tolerances are well known to constrain achievable null depths to the −60 to −80 dB range. It follows, therefore, that while the reported value of −156.3 dB serves as a meaningful benchmark for algorithmic comparison, it should not be interpreted as physically realizable performance metrics.

(ii) Constrained Sidelobe Level: For this design example of the single-nulling problem, the *MSL* was constrained to −29.75 dB as a threshold. Relative to the first design case, a gain of nearly 2 dB in *MSL* was achieved. Figure 4 presents the resulting normalized array factor. Compared to Figure 2 (unconstrained sidelobe case) and Figure 3 (deep null case), Figure 4 demonstrates that the proposed HFOSC-based optimization successfully maintains the null depth at 13.0° while significantly improving the maximum sidelobe level to −29.75 dB. This confirms that the cost function’s *EMSL* term effectively controls sidelobe degradation without sacrificing nulling performance.

(iii) Multi-Source Interference: In order to verify the efficacy and simplicity of the proposed HFOSC algorithm for multi-null steering, the design is further extended to null more than two interferers. By choosing the nulling angles θ_na_ in Equation (5), dual and triple null radiation patterns can be generated. In the case of a double null, the third and sixth sidelobe peaks of the initial pattern at 13.00° and 24.75° are successfully nulled. In the triple-null case, nulls are imposed at 13.00° (third sidelobe), 24.75° (sixth sidelobe), and 38.5° (ninth sidelobe). Figure 5 shows the dual-null pattern (13.00° and 24.75°), while Figure 6 illustrates the triple-null pattern (13.00°, 24.75°, and 38.5°). As seen in Figure 6, the proposed HFOSC method successfully creates three independent deep nulls simultaneously, with achieved null depths of −96.01 dB, −90.11 dB, and −93.33 dB, respectively. The main beam shape remains well preserved, and the maximum sidelobe level increases only moderately to −25.32 dB. This demonstrates that position-only control has sufficient degrees of freedom to handle complex multi-interferer scenarios without phase shifters.

### 4.2. Design Case II: Positional Quantization Analysis

One major challenge of nulling in the position domain alone is the precision needed for element positioning. In theoretical simulation, positions are represented as floating-point real numbers, while in real-world applications, servomotors or stepper motors operate with finite mechanical precision. To measure real-world robustness, the element locations were re-optimized using positions rounded to two decimal places for Case I (single null).

**Figure 7:**
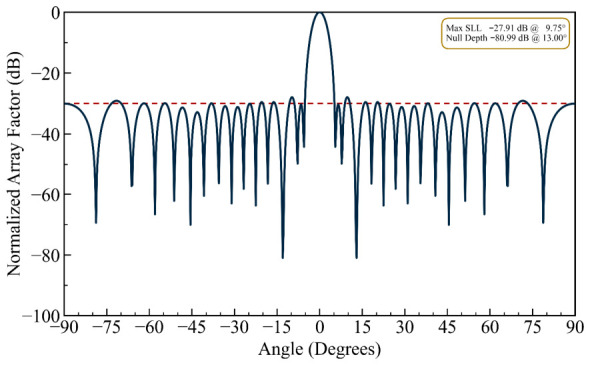
The array factor obtained from the element positions optimized to two decimal places, yielding a single null at 13.0° (the dashed line is the sidelobe envelope).

As shown in Figure 7, this results in a degradation of approximately 13 dB relative to the full-precision result, yielding a null depth of −80.99 dB. Notably, this value still falls comfortably within the −60 to −80 dB range commonly desired for effective interference cancellation, demonstrating that the algorithm retains practical utility even under positional constraints. This observation provides direct internal evidence regarding the gap between theoretical and physically realizable performance: although the optimizer achieved −156.3 dB under ideal floating-point conditions, the two-decimal-place quantization experiment shows that real-world mechanical tolerances alone are sufficient to limit the null depth to approximately −80 dB. Accordingly, the reported −156.3 dB value should be interpreted as a benchmark of the algorithm’s mathematical optimization capacity rather than a metric of physically achievable null depth in a deployed antenna array system.

### 4.3. Design Case III: Wideband Sector Nulling

**Figure 8:**
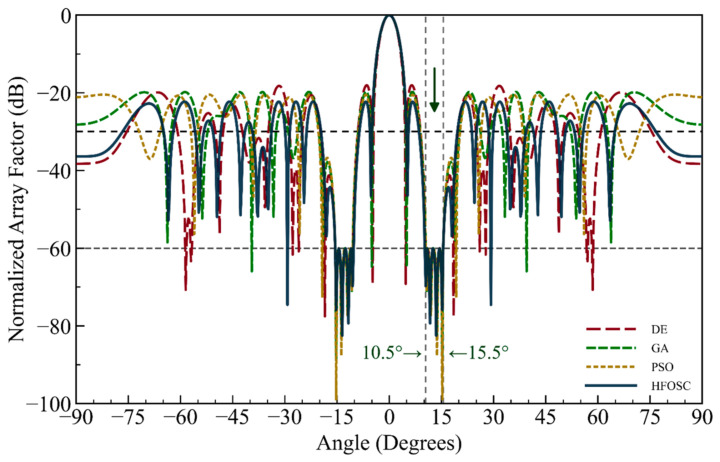
Comparison of HFOSC and conventional optimization algorithms (DE, GA, and PSO) in terms of the normalized array factor. The suppression region is centered at 13° with a 5° sector (10.5–15.5°) (the dashed line is the sidelobe envelope).

As is well known, a number of metaheuristic optimization algorithms have been proposed recently, each possessing different advantages and disadvantages, and some of them have been used to solve antenna array synthesis problems with different objectives. In the final design scenario of this paper, the well-established GA, PSO, and Differential Evolution (DE) algorithms were adapted to a wideband nulling problem and evaluated in comparison with the proposed HFOSC. For this purpose, rather than imposing an *MSL* constraint, the cost function was used to minimize the energy in a 5° wide null sector centered at 13.0° (spanning from 10.5° to 15.5°). By setting the *NDL* as −60 dB, the array element positions were obtained using conventional GA, PSO, DE, and the proposed HFOSC. As can be seen from the results in Figure 8, all algorithms satisfy the −60 dB requirement over the desired wide null region; however, the radiation pattern results of HFOSC demonstrate superior performance in terms of the *MSL* and overall pattern fidelity compared to the other algorithms.

### 4.4. Design Case IV: Practical Consideration (Mutual Coupling Effects and Position Resolution)

The previous design examples demonstrated the potential of the HFOSC-based position-only method under idealized conditions, where element positions were represented as floating-point values and the minimum inter-element spacing was at least 0.35λ. Although these results confirm the theoretical efficacy of the algorithm, real antenna arrays introduce two additional considerations: (i) the minimum allowable spacing between adjacent elements, which directly controls the magnitude of mutual coupling, and (ii) the finite mechanical resolution of physical positioning systems, which confines element locations to a discrete grid.

To investigate the performance of the proposed method under these practical constraints, a fourth design scenario is established in which the minimum inter-element spacing is increased from 0.35λ to 0.50λ. This value is widely adopted in the antenna array literature as a conservative limit below which mutual coupling effects become non-negligible and may distort both the active element patterns and the achievable null depths. By enforcing a half-wavelength floor, the optimized configurations satisfy the spacing conditions in which isolated-element pattern assumptions remain valid, ensuring that the synthesized radiation patterns are more representative of physically realizable performance.

**Figure 9:**
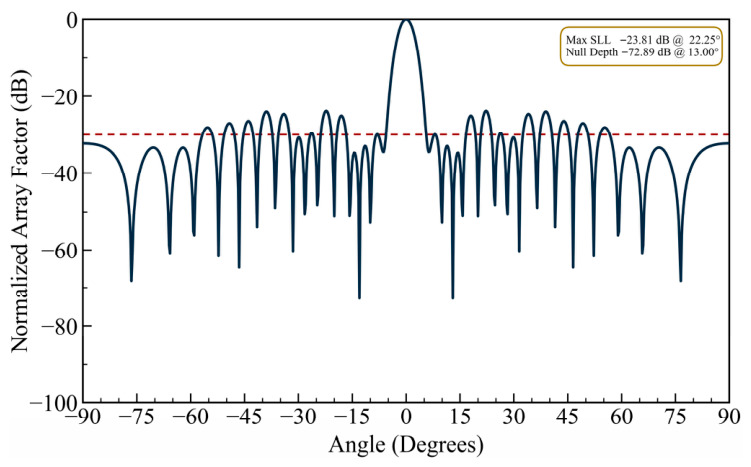
The array factor obtained under mutual coupling effects and position resolution constraints exhibiting a single null at 13.0° (the dashed line is the sidelobe envelope).

Meanwhile, the element position resolution is set to 0.1λ, which means that all optimal positions are confined to a discrete grid with a step size of one-tenth of a wavelength. This degree of quantization simulates the finite accuracy of real-world positioning actuators such as stepper motors or linear translation stages and enables a meaningful evaluation of the attainable null depth and pattern fidelity under hardware-constrained conditions. For the same single-null case of θ = 13.0°, the pattern in Figure 9 illustrates that a null depth of −72.89 dB is attained at the desired interference direction. Although this is a shallower value than the −94.29 dB obtained under floating-point accuracy with 0.35λ spacing (Design Case I), this result may still be considered adequate for interference cancellation in a practical antenna system. The highest sidelobe level is found to be −23.81 dB, which is a slight deterioration compared with the unconstrained cases, but the shape of the main beam as well as the overall sidelobe envelope of the original Chebyshev taper are still preserved.

The results of this design example illustrate that the proposed position-only null steering method retains practical utility even under realistic mutual coupling avoidance constraints and finite position resolution. The achieved null depth of about −73 dB under 0.1λ quantization with 0.5λ minimum spacing confirms that the method is capable of providing meaningful interference suppression in an operational sense, which would ease the mechanical design and reduce the risk of mutual coupling-induced pattern degradation in deployed systems.

It can be observed in the radiation patterns of Figure 2, Figure 3, Figure 4, Figure 5, Figure 6, Figure 7, Figure 8 and Figure 9 that the element positions calculated from the proposed approach are optimized successfully with good performance. The resulting array patterns meet all design specifications, including single, multiple, and extended wide nulls placed at the specified interference angles, while maintaining the main beam and the sidelobe envelope with high resemblance to the original Chebyshev distribution. In order to enable a systematic comparison, each design is analyzed herein. Figure 2, Figure 3 and Figure 4 deal with single-null configurations under different constraint regimes; the performance for two and three null positions is given in Figure 5 and Figure 6, respectively; Figure 7 quantifies the effect of positional discretization; Figure 8 provides a comparison in the case of wideband sector nulling; and Figure 9 illustrates the applicability of the method under realistic mutual coupling effects and finite position resolution constraints. What is particularly remarkable is that designers can easily trade off competing objectives such as sidelobe level, null depth, and pattern fidelity. For instance, substantially deeper nulls can be attained with minor sacrifice in position displacement and slight deterioration in sidelobe performance.

For the arrays depicted in Figure 2, Figure 3, Figure 4, Figure 5, Figure 6, Figure 7, Figure 8 and Figure 9, Table 1 presents the constant amplitude distribution of the corresponding Chebyshev pattern and the position perturbations adjusted by HFOSC, while Table 2 summarizes the performance comparison, including the null locations, achieved *NDL*s, the locations of *MSL*, and the achieved *MSL*s.

### 4.5. Limitations of the Proposed Methodology

While the numerical results and design examples presented in the preceding sections demonstrate the effectiveness of the proposed HFOSC-based position-only approach across various scenarios, it is important to recognize the assumptions and idealizations that define the scope of the current study. The main limitations of the proposed position-only methodology are related to the level of physical idealization adopted in the present study.

First, the array model is based on isotropic radiators, which simplifies the array factor formulation but does not account for the directional radiation characteristics of practical antenna elements such as microstrip patches or dipoles. Although this assumption is common in array synthesis studies and is useful for isolating the behavior of the optimization framework, it may lead to differences between the idealized and practically achievable radiation patterns, especially at larger scan angles.

Second, extremely deep nulls are achieved in some idealized numerical results to demonstrate the optimization capability of the proposed HFOSC-based position-only framework. However, in practical implementations, such extreme null depths are highly sensitive to finite position resolution, actuator tolerances, calibration errors, thermal noise, mutual coupling, element pattern deviations, manufacturing imperfections, alignment errors, and other non-ideal effects. In practice, when the null depth reaches the −50 dB to −70 dB range, the result becomes increasingly dominated by these physical and implementation-related limitations, and further numerical improvement may not translate into a comparable hardware-level benefit. Therefore, the null depth around −156 dB obtained in this work should be interpreted primarily as evidence of the mathematical search capability and idealized optimization potential of the proposed method, rather than as directly realizable performance in a physical array. The inclusion of these extremely deep-null cases is important because it shows that the proposed framework is capable of driving the solution toward exceptionally stringent minima under the assumed model, even beyond what would typically remain meaningful under practical physical constraints.

Third, the proposed method is inherently affected by mechanical positioning precision. Since the method relies exclusively on element relocation, the achievable null depth in practice is sensitive to actuator resolution and tolerance. For this reason, the manuscript includes both a finite-precision position study and the additional constrained example described above, which together provide a more realistic indication of implementation feasibility.

To partially address these practical concerns and move toward more realistic implementation conditions, both a finite-precision position study and an additional constrained example that accounts for mutual coupling have been included in Design Case IV. This example is restricted to a position resolution of 0.1λ and a minimum inter-element spacing of 0.5λ. These constraints reflect both practical actuator precision and a spacing condition chosen specifically to mitigate the influence of mutual coupling effects. The corresponding results illustrate the trade-off between achievable null depth and maximum sidelobe level under these stricter physical constraints, thereby providing a more realistic indication of implementation feasibility. In this sense, while the unconstrained results help reveal the intrinsic optimization strength of the proposed method, the constrained examples serve as a practical counterpart, demonstrating that the HFOSC-based position-only framework retains meaningful performance even under hardware-level limitations.

## 5. Comparative Performance Analysis

The proposed HFOSC algorithm is evaluated in a comparative manner in this section against commonly used metaheuristics, namely the GA, PSO, and DE. All the techniques were run under an identical problem setup, including the same antenna array structure, the same coefficient bounds, and the same wideband suppression requirement centered at 13.0° with a total suppression width of 5° (±2.5°), and the same stopping policy. Specifically, all algorithms were initialized using a uniform random population of 30 individuals and executed for a maximum of 500 iterations, with termination defined by reaching the maximum iteration limit and no early stopping mechanism applied. For statistical reliability, each algorithm was executed 100 times independently with different random initializations. The algorithm-specific parameter settings are summarized in Table 3.

For a clear assessment, the comparison is carried out from two complementary perspectives: optimization cost and convergence behavior, and radiation pattern quality metrics reflecting physical antenna performance. Table 4 presents the statistical results of the optimization cost together with the average computational time for all considered algorithms. HFOSC achieves the lowest best cost, the lowest mean cost, and the smallest standard deviation among the compared methods. These results indicate not only superior solution quality but also stable and repeatable convergence behavior across independent runs. This also suggests that HFOSC is less sensitive to random initialization and maintains a more consistent search trajectory during the optimization process. DE provides the second-best mean cost performance; however, its larger standard deviation indicates lower robustness compared with HFOSC. GA exhibits weaker overall optimization performance, with a higher mean cost and the largest average execution time. PSO attains a competitive best cost in certain runs; nevertheless, its substantially larger standard deviation reveals pronounced run-to-run variability and thus a less reliable convergence behavior. HFOSC also yields the lowest mean runtime, demonstrating that it combines high optimization accuracy with favorable computational efficiency.

Cost-based measures are informative about numerical optimization performance, but they are not sufficient to fully describe the physical quality of the resulting radiation patterns. Thus, the comparison is further supported by key electromagnetic parameters, including the HPBW, the maximum sidelobe level (*MSLL*), and the maximum *NDL*, as presented in Table 5. The HFOSC method provides the most balanced overall radiation pattern among the compared techniques, achieving the lowest mean HPBW, the strongest mean and best sidelobe suppression, and the best null-depth performance in terms of both mean and best values. In particular, the Mean Max *NDL* and Best Max *NDL* results indicate that HFOSC achieves deeper and more consistent suppression within the prescribed wideband nulling region compared with the other methods. These results demonstrate that HFOSC can enforce effective wideband suppression while preserving the main-beam characteristics more successfully than the alternative algorithms. PSO shows partially competitive behavior in some pattern metrics, but its null-depth performance remains slightly inferior to that of HFOSC. GA and DE achieve acceptable HPBW values; however, both remain weaker than HFOSC in terms of sidelobe and null suppression performance. Overall, the results in Table 5 confirm that HFOSC offers a more favorable trade-off between beam preservation and wideband null suppression.

Overall, the comparative results presented in Table 4 and Table 5 show that HFOSC delivers a more reliable and balanced optimization performance than the other metaheuristic methods considered in this study. While some competing algorithms may achieve competitive values in individual metrics, HFOSC consistently provides lower optimization cost, shorter average runtime, stronger null-depth control, and better preservation of the main-beam characteristics. These results further support the suitability of HFOSC for wideband null-steering antenna array synthesis problems. To further validate the statistical significance of the observed performance differences, the Wilcoxon rank-sum test was applied to the optimization cost results obtained from 100 independent runs. The test results confirm that the differences in optimization cost between HFOSC and each of the competing algorithms are statistically significant (*p* < 0.05), thereby providing statistical support for the superiority of the proposed algorithm.

**Figure 10:**
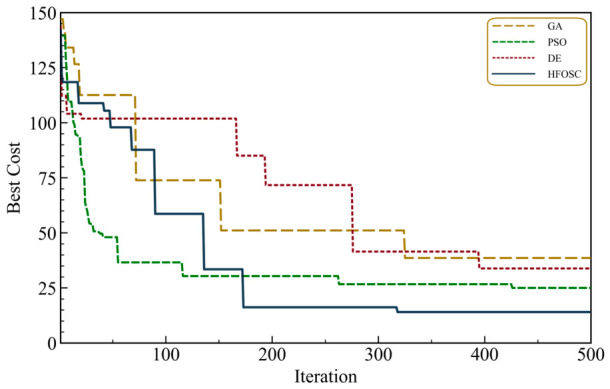
Comparative convergence performance of HFOSC, DE, GA, and PSO algorithms in terms of the best-so-far cost over independent runs.

According to Figure 10, the convergence of HFOSC in terms of the best-so-far cost over 100 independent runs is compared with those of DE, GA, and PSO. The HFOSC curve shows the best overall convergence pattern and reaches the lowest cost among the compared algorithms. Similarly, PSO has the fastest initial cost reduction and achieves the second-best convergence performance overall; however, its curve stabilizes at a higher cost level than that of HFOSC. The relative performance of GA and DE changes as the iteration proceeds. GA converges at a faster rate in the early and intermediate stages, while DE converges more gradually but eventually attains a slightly lower final cost. These results demonstrate that HFOSC achieves a better balance between exploration and exploitation, leading to improved final convergence performance and solution quality for the considered wideband null-steering problem.

## 6. Conclusions

In this paper, a design method for position-only null steering in linear antenna arrays is presented. The study considers the application of the HFOSC scheme to the synthesis of element positions for interference suppression, thereby eliminating the requirement for phase-shifting networks or variable attenuators in the beamforming architecture. In contrast to conventional metaheuristics, HFOSC exhibits three notable practical benefits: (i) faster convergence due to the maturation-driven saturation mechanism; (ii) superior run-to-run repeatability, evidenced by the smallest standard deviation among all compared optimizers; and (iii) control of both null depth and maximum sidelobe level through the proposed cost function.

The results obtained under ideal array-factor-based assumptions show that the formulation can synthesize null depths deeper than −90 dB for discrete sources and −60 dB over extended angular regions while maintaining close fidelity in the main beam and the sidelobe structure of a reference Chebyshev pattern. However, it should be emphasized that these extreme null depth values are reported primarily as indicators of the optimizer’s search capability under ideal conditions and are not intended to represent directly achievable metrics in physical hardware. Two additional design examples with a minimum inter-element spacing of 0.5λ combined with a position quantization of 0.1λ were also studied to evaluate practical feasibility; under these constraints, null depths of about −73 dB were achieved, which fall within the range typically considered practically useful. It should be noted that Design Case IV represents a single representative constrained scenario rather than a comprehensive sensitivity analysis; a systematic parametric study across a broader range of inter-element spacing values and position resolution steps would be required to fully characterize the performance envelope under hardware-constrained conditions, and this is identified as an important direction for future work. The comparison results with GA, PSO, and DE also show that HFOSC achieves more stable convergence and better null depth control for the specific problem instances considered. Taken together, these results indicate that the HFOSC-based position-only framework can serve as a viable candidate for scenarios where mechanical reconfiguration is possible and the associated implementation constraints can be accommodated.

As future work, element radiation patterns should be made more realistic, and mutual coupling should be modeled explicitly through full-wave electromagnetic simulation, with results validated experimentally using a prototype positioning system. Such studies would help to bridge the gap between the algorithmic synthesis presented here and the deployment of mechanically reconfigured antenna arrays in real-world next-generation wireless communication systems.

## Figures and Tables

**Table 1:** Chebyshev Excitation Amplitudes, Initial Element Positions, and HFOSC-Optimized Position Perturbations (δ_n_, in λ) for the Design Scenarios in Figure 2, Figure 3, Figure 4, Figure 5, Figure 6, Figure 7, Figure 8 and Figure 9.

Index	Initial Chebyshev Pattern		Element Position Perturbations (δ_n_) in λ Obtained by Using HFOSC Algorithm							
n	Amp.	Pos.	Figure 2	Figure 3	Figure 4	Figure 5	Figure 6	Figure 7	Figure 8	Figure 9
±1	1.000000	0.25	−0.013162	−0.011064	−0.007520	0.005012	−0.014492	−0.01	0.010371	0.0
±2	0.986967	0.75	−0.035705	−0.035806	−0.020533	−0.025090	−0.036019	−0.03	0.036642	0.0
±3	0.961307	1.25	−0.033076	−0.033837	−0.023883	−0.062044	−0.034159	−0.04	0.114272	0.0
±4	0.923809	1.75	−0.028798	−0.036741	−0.015996	−0.053205	−0.040857	−0.03	0.164127	0.0
±5	0.875622	2.25	−0.006946	−0.003266	0.004274	−0.001787	−0.006733	−0.01	0.138133	0.0
±6	0.818194	2.75	0.026978	0.030030	0.024380	0.052978	0.076865	0.01	0.182293	0.0
±7	0.753225	3.25	0.057082	0.039821	0.039012	0.045842	0.079782	0.04	0.077182	0.0
±8	0.682586	3.75	0.042600	0.033751	0.038791	0.016785	0.002654	0.04	0.043665	0.1
±9	0.608252	4.25	0.032628	0.032016	0.021983	−0.005799	0.006866	0.02	0.025684	0.2
±10	0.532218	4.75	−0.017485	−0.041190	−0.013663	−0.016064	−0.004458	−0.01	0.136478	0.2
±11	0.456427	5.25	−0.020549	−0.028336	−0.045862	0.005187	0.000622	−0.04	−0.008640	0.2
±12	0.382698	5.75	−0.055758	−0.064398	−0.067293	−0.029940	0.025048	−0.07	−0.115479	0.3
±13	0.312661	6.25	−0.017362	−0.033166	−0.057235	−0.036519	0.004589	−0.04	−0.260557	0.4
±14	0.247706	6.75	0.014339	−0.020939	0.001389	−0.057767	−0.029727	−0.04	−0.375142	0.8
±15	0.423453	7.25	0.052190	0.046484	0.135600	0.065050	0.101643	0.08	0.378654	0.8

**Table 2:** Null Steering Performance Summary: Achieved Null Depths, Sidelobe Levels, and Null Locations Across Eight Design Scenarios (Figure 2, Figure 3, Figure 4, Figure 5, Figure 6, Figure 7, Figure 8 and Figure 9).

Metric	Single Null (Figure 2)	Deep Null (Figure 3)	Low Sidelobe (Figure 4)	Dual Null (Figure 5)	Triple Null (Figure 6)	Trun. Null (Figure 7)	Wide Null (Figure 8)	Pos. Const. (Figure 9)
Null Location(s) (degree)	13.00°	13.00°	13.00°	13.00° 24.75°	13.00° 24.75° 38.50°	13.00°	10.5–15.5°	13.00°
Achieved NDL(s) (dB)	−94.29	−156.25	−90.18	−94.43 −93.99	−96.01 −90.11 −93.33	−80.99	<−60.100	−72.89
Location of MSL (degree)	9.75°	90.00°	6.50°	16.50°	16.50°	9.75°	22.00°	22.50°
Achieved MSL (dB)	−27.71	−26.95	−29.75	−26.41	−25.32	−27.91	−22.29	−23.81

**Table 3:** Parameter settings of the optimization algorithms used in the comparative analysis.

Parameter	HFOSC	GA	PSO	DE
Maturation period	15	—	—	—
Mixing probability	0.50	—	—	—
Saturation threshold	3	—	—	—
Crossover rate	—	0.70	—	0.90
Mutation rate	—	0.15	—	—
Selection method	—	Tournament (k = 2)	—	—
Elitism	—	5%	—	—
Inertia weight (w)	—	—	0.3	—
Cognitive coef. (c1)	—	—	0.52	—
Social coef. (c2)	—	—	0.52	—
Scaling factor	—	—	—	0.50
Strategy	—	—	—	Rand/1/bin

**Table 4:** Statistical comparison of optimization cost and computational performance over 100 independent runs.

Algorithm	Best Cost	Mean Cost	Std Cost	Mean Time (s)
DE	33.82	37.41	15.64	4.16
GA	38.57	49.26	17.83	4.87
PSO	25.01	55.89	28.96	3.91
HFOSC	14.03	19.38	9.10	3.74

**Table 5:** Comparison of radiation pattern performance metrics obtained by different optimization algorithms.

Algorithm	Mean HPBW (deg)	Mean *MSLL* (dB)	Best *MSLL* (dB)	Mean Max *NDL* (dB)	Best Max *NDL* (dB)
DE	4.57	−20.47	−21.14	−59.75	−60.07
GA	4.51	−19.76	−20.94	−59.46	−60.05
PSO	4.66	−19.55	−20.46	−59.23	−60.01
HFOSC	4.27	−21.41	−22.29	−60.02	−60.10

## Data Availability

All data generated or analyzed during this study are included in the manuscript.
